# Valuable Contempt

**Published:** 1905-02

**Authors:** 


### Valuable Contempt.

On September 30th Dr. W. A. Whitlock, of Merced, was called as a witness in the superior court to give evidence in a case there pending. He gave all of the general evidence asked, but when the attorney for the defendant asked for expert evidence relating to certain facts about a gunshot wound, Dr. Whitlock refused to answer, on the ground that such testimony was expert evidence, and that the witness should receive proper compensation for his time. The court adjudged him in contempt, and sent him to jail, as he persisted in refusing to answer the questions. Later the district attorney visited him in jail and agreed to approve his claim for $5 if he would consent to testify. This he did, and was released. Dr. Whitlock says: "I went to jail to protect the rights of the profession, and would have been there yet if the district attorney had not come to my terms." Certainly, if the facts as reported to the *Journal* are correct, and we have no reason to doubt them, the physicians of this State are indebted to Dr. Whitlock for his courage in sticking up for his rights and the rights of every expert witness.—*Calif. Journal Med.*

